# Fasudil, a Rho-Kinase Inhibitor, Attenuates Bleomycin-Induced Pulmonary Fibrosis in Mice

**DOI:** 10.3390/ijms13078293

**Published:** 2012-07-04

**Authors:** Chunguo Jiang, Hui Huang, Jia Liu, Yanxun Wang, Zhiwei Lu, Zuojun Xu

**Affiliations:** 1 Department of Respiratory Medicine, Peking Union Medical College Hospital, Chinese Academy of Medical Sciences & Peking Union Medical College, Beijing 100730, China; E-Mails: jiang.cg@hotmail.com (C.J.); pumchhh@126.com (H.H.); liujiafree@163.com (J.L.); wangyanxun2008@163.com (Y.W.); 2 Department of Respiratory Medicine, Yijishan Hospital of Wannan Medical College, Wuhu 241001, China; E-Mail: pumc-luzhiwei@hotmail.com

**Keywords:** pulmonary fibrosis, fasudil, transforming growth factor-β1, connective tissue growth factor, plasminogen activator inhibitor-1

## Abstract

The mechanisms underlying the pathogenesis of idiopathic pulmonary fibrosis (IPF) involve multiple pathways, such as inflammation, epithelial mesenchymal transition, coagulation, oxidative stress, and developmental processes. The small GTPase, RhoA, and its target protein, Rho-kinase (ROCK), may interact with other signaling pathways known to contribute to pulmonary fibrosis. This study aimed to determine the beneficial effects and mechanisms of fasudil, a selective ROCK inhibitor, on bleomycin-induced pulmonary fibrosis in mice. Our results showed that the Aschcroft score and hydroxyproline content of the bleomycin-treated mouse lung decreased in response to fasudil treatment. The number of infiltrated inflammatory cells in the bronchoalveolar lavage fluid (BALF) was attenuated by fasudil. In addition, fasudil reduced the production of transforming growth factor-β1 (TGF-β1), connective tissue growth factor (CTGF), alpha-smooth muscle actin (α-SMA), and plasminogen activator inhibitor-1 (PAI-1) mRNA and protein expression in bleomycin-induced pulmonary fibrosis. These findings suggest that fasudil may be a potential therapeutic candidate for the treatment of pulmonary fibrosis.

## 1. Introduction

Idiopathic pulmonary fibrosis (IPF) is a specific form of chronic, progressive fibrosing interstitial lung disease of unknown etiology [1]. The mechanisms underlying the pathogenesis of IPF involve multiple pathways, such as inflammation, epithelial mesenchymal transition, coagulation, oxidative stress, and developmental processes, which result in alveolar epithelial cell injury and fibroblast proliferation that consequently leads to abnormal deposition of extracellular collagen [2]. To date, there are no effective treatments for IPF, and IPF remains a fatal disorder. The 3- and 5-year mortality rates are approximately 50 and 80%, respectively, in the absence of lung transplantation [3]. Therefore, novel therapeutic agents for this unmet medical need are of particular interest.

The small GTPase, RhoA, belongs to the Rho subfamily and has been implicated in many cellular functions, such as cell adhesion, cell motility and migration, growth control, cell contraction, and cytokinesis. One of its main effectors, Rho-kinase (ROCK), has two known isoforms (ROCK1 and ROCK2) and regulates cytoskeletal reorganization by phosphorylating myosin phosphatase, which results in an increase in myosin light chain (MLC) phosphorylation [4]. The Rho/ROCK-mediated pathway plays a role in infiltration of inflammatory cells both *in vitro* and *in vivo* [5,6]. Transforming growth factor-β1 (TGF-β1) links inflammation to fibrogenesis and is one of the key mediators in the fibrotic process [7]. As a downstream mediator of TGF-β1, connective tissue growth factor (CTGF) plays a crucial role in TGF-β-induced connective tissue cell proliferation and extracellular matrix deposition [8]. Watts *et al.* previously demonstrated that both CTGF overexpression and myofibroblast formation in idiopathic pulmonary fibrosis cell lines are dependent on RhoA signaling [9]. The authors also showed that cyclin D1 expression is deregulated in idiopathic pulmonary fibrosis through a RhoA-dependent mechanism that influences lung fibroblast proliferation [10]. Because the Rho/ROCK-mediated pathway might interact with other signaling pathways known to contribute to pulmonary fibrosis [5–11], we hypothesized that fasudil, which is a highly selective inhibitor of both ROCK isoforms, may inhibit the development of pulmonary fibrosis.

Intratracheal administration of bleomycin (Bleo) is the most extensively used experimental model of pulmonary fibrosis, since the features of pathogenesis are very similar to IPF [12]. Using this model, we investigated the inhibitory effect of fasudil on IPF. We evaluated histological findings of bleomycin-induced pulmonary fibrosis by determining the fibrotic score and measuring hydroxyproline content in the lungs. To elucidate the mechanisms of fasudil-induced inhibition of the pulmonary fibrosis model, the number of inflammatory cells in bronchoalveolar lavage fluid (BALF) as well as TGF-β1, CTGF, alpha-smooth muscle actin (α-SMA), and plasminogen activator inhibitor-1 (PAI-1) mRNA and protein levels in the lungs were examined. In addition, we measured total myosin phosphatase targeting subunit 1 (MYPT1) phosphorylation levels of lung homogenates from the mice.

## 2. Results

### 2.1. Effect of Fasudil on Histopathology

**Figure 1:**
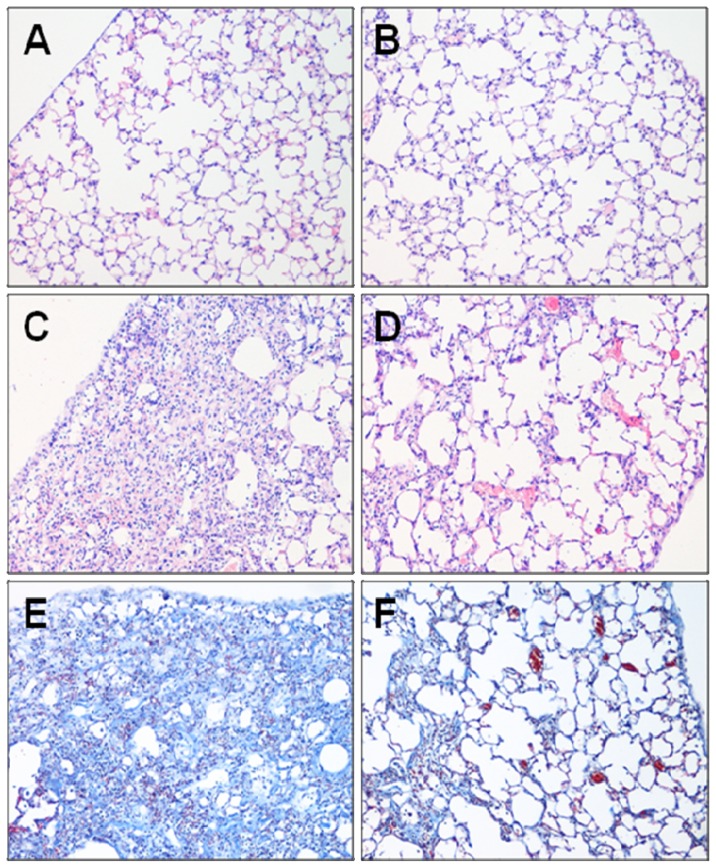
Representative histological lung sections from each group. Removed lungs on day 21 after bleomycin administration were stained with Hematoxylin-eosin (**A**–**D**) or Masson-trichrome stain (**E** and **F**) (magnification: ×100). (A) PBS+NS group, (B) PBS+FSH group, (C and E) Bleo+NS group, and (D and F) Bleo+FSH group.

The effect of fasudil against bleomycin-induced inflammation and fibrosis was examined on day 21 after bleomycin infusion (Figure 1). A well-alveolized normal histology was observed in the phosphate-buffered saline (PBS) + normal saline (NS)-treated control group. No morphological changes were observed in the PBS + fasudil at 100 mg/kg body weight (FSH)-treated group either. In contrast, bleomycin stimulation induced obvious alveolar wall thickening, massive infiltration of leukocytes, and excessive deposition of mature collagen in the interstitium. Although fibrotic lesions were observed in the Bleo + FSH-treated group, both the extent and intensity of the lesions were less than those of the Bleo + NS-treated group.

**Figure 2:**
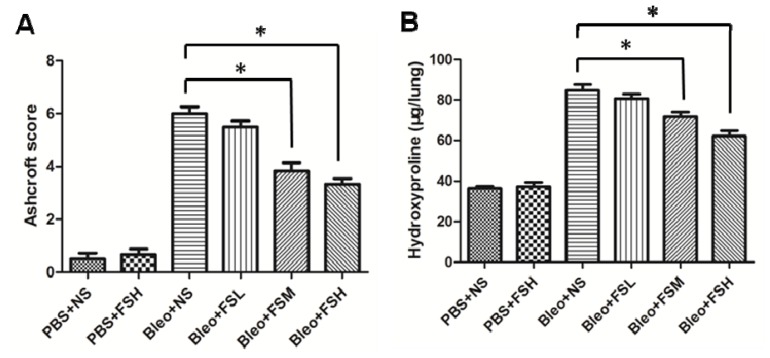
Evaluation of fibrotic changes by Ashcroft score (**A**) and hydroxyproline content (**B**) on day 21 after bleomycin administration. Results are expressed as means ± standard error of the mean (SEM) (*n* = 6). Statistical analysis was performed using one-way analysis of variance (ANOVA) followed by Tukey’s multiple comparison test (* *p* < 0.05).

To confirm the effect of fasudil on the histopathological change of bleomycin-induced pulmonary fibrosis, the overall grades of the fibrotic changes in the lungs were determined using the Ashcroft scoring method (Figure 2A). Scores of the PBS + NS-treated group and PBS + FSH-treated group were 0.50 ± 0.22 and 0.67 ± 0.21, respectively. The fibrotic scores in the Bleo + NS, fasudil at 1 mg/kg body weight (FSL), fasudil at 10 mg/kg body weight (FSM), and FSH-treated groups were 6.00 ± 0.26, 5.50 ± 0.22, 3.83 ± 0.31, and 3.33 ± 0.21, respectively. Bleomycin administration induced a significant increase in the fibrotic scores compared to controls (*p* < 0.05). Importantly, the scores of the mice administered 10 and 100 mg/kg fasudil were significantly suppressed (*p* < 0.05). However, the higher dose of fasudil was not associated with a more significant reduction in the Ashcroft score (*p* > 0.05).

### 2.2. Effect of Fasudil on Hydroxyproline Level

To quantitatively assess the difference in the extent of pulmonary fibrosis in the bleomycin-treated mice with or without fasudil, we measured the hydroxyproline content on day 21 after bleomycin infusion (Figure 2B). The hydroxyproline content in the PBS + NS-treated group was 36.32 ± 1.08 mg/lung. The hydroxyproline content in the Bleo + NS, FSL, FSM, and FSH-treated groups was 84.79 ± 3.04, 80.73 ± 2.21, 71.80 ± 2.33, and 62.18 ± 2.75 mg/lung, respectively. Administration of FSH alone did not affect hydroxyproline content (*p* > 0.05), but bleomycin administration induced a significant increase in the level of hydroxyproline (*p* < 0.05). The amount of hydroxyproline was significantly decreased in the mice administered 10 and 100 mg/kg fasudil (*p* < 0.05). The higher dose of fasudil was not associated with a more significant reduction in hydroxyproline content (*p* > 0.05).

### 2.3. Effect of Fasudil on Infiltration of the Inflammatory Cells in Airways

**Figure 3:**
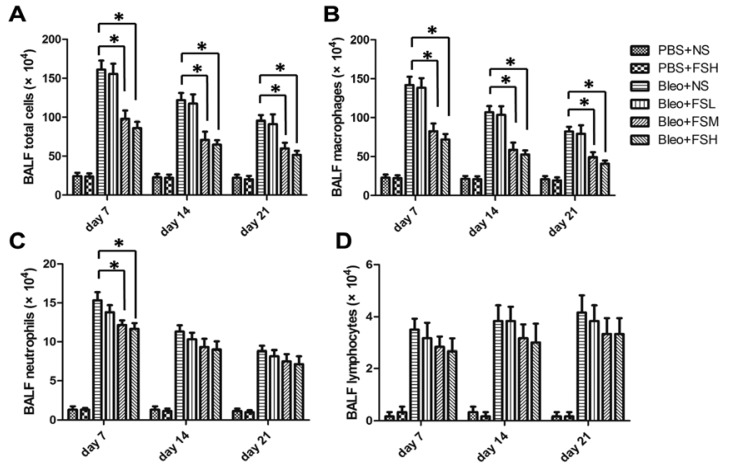
Infiltration of inflammatory cells in BALF collected on days 7, 14 and 21 after bleomycin administration. (**A**) Total cell numbers; (**B**) macrophage numbers; (**C**) neutrophil numbers; and (**D**) lymphocyte numbers, respectively. Results are expressed as means ± SEM (*n* = 6). Statistical analysis was performed using one-way ANOVA followed by Tukey’s multiple comparison test (* *p* < 0.05).

To evaluate the effect of fasudil on bleomycin-induced infiltration of inflammatory cells into the airways, we counted the number of total cells, alveolar macrophages, neutrophils, and lymphocytes in BALF on days 7, 14, and 21 after bleomycin treatment (Figure 3). Intratracheal bleomycin administration induced a significant increase in the number of inflammatory cells (*p* < 0.05). Treatment with 10 and 100 mg/kg fasudil significantly reduced the total number of cells and macrophages from day 7 to day 21, and neutrophils by day 7 (*p* < 0.05), but did not affect the number of lymphocytes at any of the time points assessed (*p* > 0.05). The higher dose of fasudil was not associated with a more significant reduction in the numbers of total cells, neutrophils, or lymphocytes (*p* > 0.05).

### 2.4. Effect of Fasudil on TGF-β1, CTGF, α-SMA, and PAI-1

**Figure 4:**
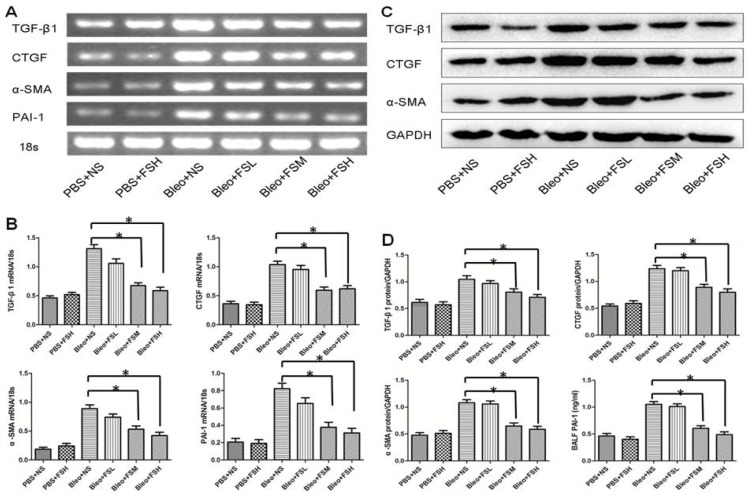
The levels of TGF-β1, CTGF, α-SMA and PAI-1 mRNA expression and protein in the lung on day 21 after bleomycin administration. The mRNA expression was examined in lung tissue by semi quantitative RT-PCR, protein level of TGF-β1, CTGF and α-SMA by Western blotting, while the level of PAI-1 protein in BALF was measured by ELISA. Results are expressed as means ± SEM (*n* = 6). Statistical analysis was performed using one-way ANOVA followed by Tukey’s multiple comparison test (* *p* < 0.05).

A variety of fibrogenic cytokines have been implicated in the development of bleomycin-induced pulmonary fibrosis, including TGF-β1, CTGF, α-SMA, and PAI-1 [13–16]. To assess the effects of fasudil on TGF-β1, CTGF, α-SMA, and PAI-1 production, the gene expression of TGF-β1, CTGF, α-SMA, and PAI-1 were examined in lung tissue by semi quantitative RT-PCR, and the protein level of TGF-β1, CTGF, and α-SMA by western blotting, respectively, while the level of PAI-1 protein in BALF was measured by ELISA on day 21 after bleomycin infusion (Figure 4). Intratracheal bleomycin administration induced a significant increase in the levels of TGF-β1, CTGF, α-SMA and PAI-1 mRNA and protein (*p* < 0.05). Treatment with 10 and 100 mg/kg fasudil significantly decreased the levels of TGF-β1, CTGF, α-SMA, and PAI-1 mRNA and protein compared to the bleomycin alone group (*p* < 0.05). The higher dose of fasudil was not associated with a more significant reduction in the level of TGF-β1, CTGF, α-SMA, and PAI-1 mRNA and protein (*p* > 0.05). Importantly, fasudil treatment alone did not affect the expression of these genes in the lung at the mRNA or protein level compared to the controls (*p* > 0.05).

### 2.5. MYPT1 Phosphorylation Levels in Mouse Lungs

**Figure 5:**
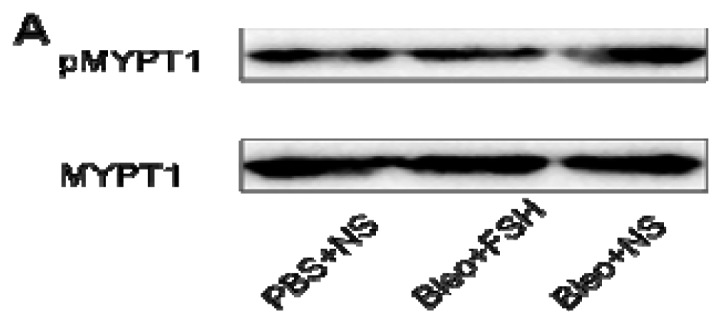

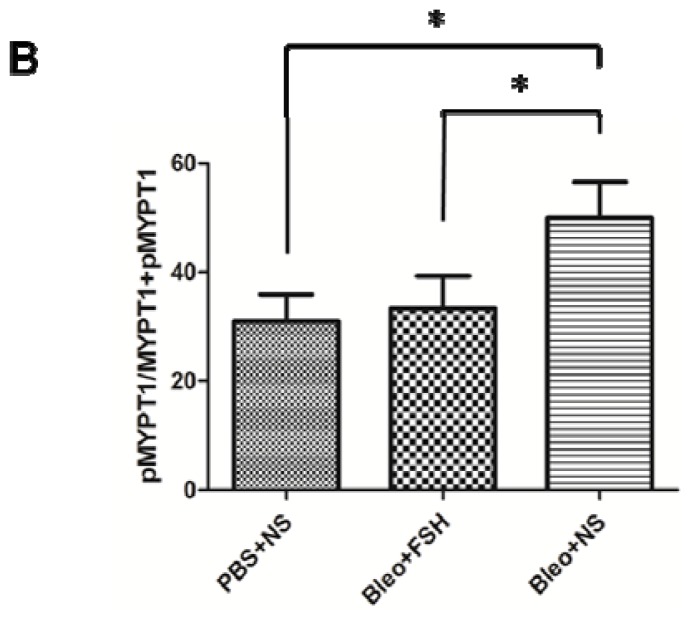
MYPT1 phosphorylation in the lung on day 21. The relative amounts of MYPT1 phosphorylation are expressed as pMYPT1/MYPT1+pMYPT1. Results are expressed as means ± SEM (*n* = 6). Statistical analysis was performed using one-way ANOVA followed by Tukey’s multiple comparison test (* *p* < 0.05).

The level of MYPT1 phosphorylation in the lung was analyzed by Western blot on day 21 after intratracheal administration of bleomycin (Figure 5). Intratracheal bleomycin administration induced a significant increase in the level of MYPT1 phosphorylation (*p* < 0.05). Treatment with 100 mg/kg fasudil significantly decreased the level of MYPT1 phosphorylation in the lungs of bleomycin-treated mice (*p* < 0.05).

## 3. Discussion

To our knowledge, this is the first report of an intraperitoneal application of fasudil, a ROCK inhibitor, for the attenuation of pulmonary fibrosis induced by bleomycin in mice. In this study, fasudil administration significantly (1) improved histopathological features and decreased hydroxyproline content in the lungs of bleomycin-treated mice; (2) attenuated infiltration of inflammatory cell numbers in the BALF; and (3) reduced the production of TGF-β1, CTGF, α-SMA, and PAI-1 mRNA and protein expression in bleomycin-induced pulmonary fibrosis.

Intratracheal instillation of bleomycin induces acute lung inflammation with infiltration of inflammatory cells [6]. Among inflammatory cells, neutrophilia in the lungs worsens the prognosis of IPF and attenuates the response to corticosteroids in humans [17]. These inflammatory cells can synthesize and secrete various cytokines, chemokines, reactive oxygen species, and proteases, which can lead to aberrant fibroproliferation and collagen production in mice [18]. In the present study, the number of neutrophils and macrophages in the BALF was significantly decreased by administration of fasudil to bleomycin-treated mice. Interleukin-1 (IL-1) is a pivotal inflammatory cytokine that can activate RhoA and in turn evokes Rho-dependent cytoskeletal reorganization *in vitro* [19]. Shimizu and colleagues have demonstrated that Y-27632, another Rho-kinase inhibitor, can inhibit IL-1β-induced neutrophil migration in a dose-dependent manner [6]. ROCK up-regulates molecules, including nuclear factor-kappa B (NF-κB) [20], nicotinamide adenine dinucleotide phosphate (NAD(P)H) [21], IL-6 [22], endothelial nitric oxide synthase (eNOS) [23], monocyte chemoattractant protein-1 (MCP-1) [24], macrophage migration inhibitory factor (MMIF), and interferon-γ [25], all of which are involved in the pathogenesis of inflammation. In the current study, we were not able to determine the exact mechanism responsible for these observations. However, our findings suggest that the inhibitory effects of fasudil on the inflammation response may be one of the reasons of its anti-fibrotic effect. The inflammation response occured in the early stage of the disease and then gradually reduced. Until now, anti-inflammatory strategies aimed at preventing the fibroproliferative response in IPF have been unsuccessful [26]. In the current study this also implies a limited beneficial effect of fasudil during the late stage of the disease.

Evidence from human studies and animal models indicates that TGF-β1 is an important mediator with a broad spectrum of activities in pulmonary inflammation, tissue repair, and fibrosis [27–29]. The up-regulation of TGF-β1 and CTGF play critical roles in the pathogenesis of bleomycin-induced pulmonary fibrosis [13,14,30]. In our study, TGF-β1 and CTGF production in the lungs were elevated after bleomycin treatment, which was consistent with previous investigations [31,32]. However, the increased TGF-β1 and CTGF production was reduced by fasudil treatment. CTGF acts downstream and in concert with TGF-β1 to drive fibrogenesis. Watts and colleagues previously demonstrated that simvastatin can modulate CTGF expression and the interaction with TGF-β1 through a Rho signaling mechanism in lung fibroblasts [9]. Collectively, these findings indicate that the fasudil-mediated attenuation of pulmonary fibrosis may be at least partially attributed to its inhibitory effect on fibrogenic cytokines production, which thereby modulates collagen synthesis.

Epithelial-to-mesenchymal transition (EMT) plays an important role in the fibrotic response and α-SMA is considered to be a characteristic marker of myofibroblasts [15]. A previous study reported that inhibition of the Rho/ROCK pathway on chronic allograft nephropathy in rats might antagonize the process of EMT [33]. Another study reported that fasudil may reduce EMT and renal interstitial fibrosis in diabetic rats through a mechanism by which ROCK activity is inhibited [34]. Moreover, TGF-β1 has been shown to be an important mediator of pulmonary fibrosis and can induce differentiation of pulmonary fibroblasts into myofibroblasts, which is characterized by α-SMA expression and active synthesis of extracellular matrix (ECM) proteins [35,36]. In the present study, fasudil attenuated α-SMA mRNA and protein expression in bleomycin-treated lungs. Therefore, we assumed that the effect of fasudil on bleomycin-induced pulmonary fibrosis may be partly due to the suppression of EMT.

It is well known that fibrin deposits persist in patients with fibrotic lung diseases because the normal fibrinolytic activity is suppressed by an increased expression of plasminogen activator inhibitor-1 (PAI-1) [37–39]. PAI-1-deficient mice develop less fibrosis and survive longer after bleomycin exposure compared to wild-type mice, while transgenic mice overexpressing PAI-1 develop more severe bleomycin-induced pulmonary fibrosis [40]. Repeated administration of small interfering RNA (siRNA) that targets PAI-1 in the lung has been shown to attenuate the development and progression of pulmonary fibrosis [16]. Many studies have tried to link PAI-1 with RhoA activity. Nishikimi *et al.* revealed that the Rho/Rho-kinase pathway participates in the pathogenesis of nephrosclerosis in part through the up-regulation of PAI-1 [41]. Ishimaru *et al.* reported that fasudil attenuates myocardial fibrosis possibly through suppression of PAI-1 in deoxycorticosterone acetate (DOCA)-salt hypertensive rats [42]. In this study, our data demonstrated that the levels of PAI-1 mRNA and protein were markedly lower in mice treated with fausdil and bleomycin than those treated with bleomycin without fasudil. Therefore, we hypothesize that the effect of fasudil on bleomycin-induced pulmonary fibrosis may be partially caused by inhibition of PAI-1.

A variety of pharmacological agents have been proposed as therapies for pulmonary fibrosis; however, there are currently no effective treatments [1]. Corticosteroids and cyclophosphamide are widely used as standard protocols of current therapy in IPF, but the effects are not optimal for universal use [1]. In addition, corticosteroids block a broad array of pathways and thereby cause several side effects, including an increased risk of infection. In the present study, our findings indicate that fasudil may become a novel potential treatment for IPF. In addition, fasudil has been used as a vasodilator for a long period of time and has few side effects, with no serious adverse events reported to date [43]. However, additional studies are needed to further explore the use of fasudil for this indication. First, we administered fasudil starting on day 0 post-bleomycin treatment, and therefore additional studies are necessary to test for a reversal effect of fasudil in a time-dependent manner, such as at 7 d post-bleomycin injection, which is the time point at which pneumonitis and fibrosis are established. Second, although inhibition of the recruitment of inflammatory cells as well as TGF-β1, CTGF, α-SMA, and PAI-1 expression were observed during fasudil administration, the detailed mechanisms by which fasudil acts to prevent bleomycin-induced pulmonary fibrosis remain to be determined in the context of the pleiotropic effects of fasudil and the complex pathogenesis of pulmonary fibrosis. Third, although high dose fasudil treatment has normalized MYPT1 phosphorylation completely, there were still differences in the extent of pulmonary fibrosis between high dose fasudil treatment and control group. The mechanism of pulmonary fibrosis is so perplexing that Rho/ROCK may be just one of several important pathways. In addition, further studies are required to clarify whether the long-term administration of fasudil would be beneficial for patients with lung fibrotic diseases.

## 4. Experimental Section

### 4.1. Study Design and Experimental Protocol

Pathogen-free male C57BL/6 mice (6–8-weeks old; 20–25 g body weight) were used for the experiments according to international and institutional guidelines for animal care. The study was approved by the Animal Ethics Committee of Peking Union Medical College Hospital. Clean water and rodent laboratory food were supplied under standard conditions *ad libitum*.

Mice were anesthetized with pentobarbital by intraperitoneal (i.p.) injection. Bleomycin sulfate (Nippon Kayaku, Japan) diluted in 50 μL of sterile PBS was then administered through a single intratracheal injection at a dose of 2.5 mg/kg body weight on day 0. Fasudil (Asahi Kasei, Japan) at 1, 10, and 100 mg/kg body weight dissolved in 100 mL sterile, NS was given by i.p. injection once a day starting on day 0, and was continued until the point at which the mice were euthanized. Control mice were injected with NS in a similar manner. BALF was collected to determine cell differentiation on days 7, 14, and 21 after the initial injection of bleomycin. Mice were anesthetized with an injection of pentobarbital and the BALF procedure was performed. After collecting the BALF samples, the lungs were perfused with 5 ml of cold saline through the left ventricle and surgically removed. The left lungs were used to evaluate the fibrotic score by histological examination, and the right lungs were then homogenized to analyze the expression of hydroxyproline content as well as TGF-β1, CTGF, α-SMA, and PAI-1 mRNA and protein expression levels. The MYPT-1 phosphorylation level was also assessed.

One hundred and eight mice were randomly allocated into the following six experimental groups: 1. PBS + NS; 2. PBS + FSH; 3. Bleo + NS; 4. Bleo + FSL; 5. Bleo + FSM; and 6. Bleo + FSH.

### 4.2. Histopathological Examination

The the lungs were excised on day 21 after bleomycin infusion and immediately fixed with 4% paraformaldehyde for 48 h, embedded in paraffin, and then sectioned at a 5 μM thickness. The sections were stained with hematoxylin and eosin (H&E) and Masson-trichrome. Histopathological scoring of pulmonary fibrosis was performed according to the method reported by Ashcroft *et al*. [44]. The grade of fibrotic changes in each lung section was assessed as a mean score of severity from at least 10 randomly chosen high-power fields. To minimize bias of observation, all histological specimens were randomly numbered and the grading was performed in a blinded manner by two independent, experienced pathologists.

### 4.3. Bronchoalveolar Lavage Fluid (BALF)

The BALF procedure was conducted on days 7, 14, and 21 after bleomycin administration as previously described [45]. Briefly, the trachea was exposed and cannulated with a 20-gauge catheter. After instillation of 0.8 mL of cold sterile PBS three times through the trachea into the lung, BALF was recovered at 90% of the original volume. The BALF was centrifuged for 10 min at 1500 rpm and the cell-free supernatant was stored at −70 °C. The total number of cells in BALF was counted using a hemocytometer. Then BALF was spun onto microscope slides using cytospin for 5 min at 800 rpm and routinely stained. The percentage of macrophages, neutrophils, and lymphocytes was calculated by counting 200 cells on randomly selected sections of the slide based on morphology.

### 4.4. Enzyme-Linked Immunosorbent Assay

The concentrations of PAI-1 in BALF were determined using an enzyme-linked immunosorbent assay (ELISA) kit (Yanhui, China) on day 21 after bleomycin instillation. The procedure was performed according to the manufacturer’s instructions.

### 4.5. Hydroxyproline Assay

Total lung collagen was determined by analysis of hydroxyproline on day 21 after bleomycin infusion as previously described [46]. In brief, the right upper lobes of the lung were homogenized in 12 N HCl and hydrolyzed at 120 °C overnight. Citrate/acetate buffer (pH 6.0) and chloramine T solution were added at room temperature for 20 min. The mixture was then incubated with Ehrlich’s solution at 65 °C for 15 min. After the samples were cooled to room temperature, the absorbance of each sample at 550 nm was measured. Hydroxyproline content was calculated from a standard curve of hydroxyproline (Sigma, MO, USA) and expressed as μg hydroxyproline per mg wet lung weight.

### 4.6. RT-PCR

Total RNA was extracted from the lung using Trizol reagent (Invitrogen, CA, USA) according to the manufacturer’s instructions. RNA was reverse-transcribed into cDNA using M-MuLV reverse transcriptase (Invitrogen, CA, USA). PCR was then performed in a final volume of 20 μL using a PCR reagent kit (Takara, Shiga, Japan). The 18s gene was used as an internal control. The cycling program involved preliminary denaturation at 95 °C for 10 min, followed by 35 cycles of 94 °C for 30 s, 55 °C for 30 s, and 72 °C for 1 min, followed by a final elongation step at 72 °C for 5 min. The sequences of the primers and products are listed in Table 1.

### 4.7. Western Blot

The lungs were cut with scissors into fragments of 0.5–2.0 mm^3^ and then ground in a glass homogenizer on ice. The lung tissue was lysed with RIPA lysis buffer (Beyotime, China) for 30 min and centrifuged. The protein concentration was determined with the Bradford protein assay reagent (Beyotime, China). Total protein for each sample (50 μg) was resolved by 10% sodium dodecyl sulfate-polyacrylamide gel electrophoresis (SDS-PAGE) and transferred onto a nitrocellulose membrane (Amersham Pharmacia Biotech, Buckinghamshire, UK). After blocking with 5% skim milk in Tween 20 (Beyotime, China) at room temperature for 1 h, the membrane was incubated with primary rabbit polyclonal anti-TGF-β1 antibody (1:500; Santa Cruz, CA, USA), anti-CTGF antibody (2 μg/mL; Biovision, CA, USA), anti-pMYPT1 antibody (1:500; Cell Signaling Technology, USA), anti-MYPT1 antibody (1:500; Cell Signaling Technology, USA), anti-GAPDH antibody (1:500; Santa Cruz, CA, USA), or mouse monoclonal anti-α-SMA antibody (1:1000; Santa Cruz, CA, USA) at 4 °C overnight. After washing with Tris-buffered saline (TBS; Beyotime, China), the membrane was incubated with horseradish peroxidase (HRP)-conjugated goat anti-rabbit or anti-mouse IgG antibody (1:10,000; Santa Cruz, CA, USA) for 1 h at room temperature. After washing with TBS, the bound antibody was visualized according to standard protocols for the electrochemiluminescence (ECL) detection system (Amersham, NY, USA).

### 4.8. Statistical Analysis

The results were presented as means ± SEM. Comparisons were performed using one-way ANOVA followed by Tukey’s multiple comparison test (Graph Pad Software, CA, USA). A *p* value < 0.05 was considered statistically significant.

## 5. Conclusions

In summary, the present study has shown that treatment with a Rho-kinase inhibitor, fasudil, attenuates bleomycin-induced pulmonary fibrosis in mice. The beneficial effect of fasudil was possibly related to the inhibition of inflammatory cell (predominantly neutrophils and macrophages) recruitment and decrease in the production of TGF-β1, CTGF, α-SMA, and PAI-1. Our findings suggest that fasudil may be a potential therapeutic candidate in the treatment of pulmonary fibrosis.

## Figures and Tables

**Table 1:** RT-PCR primers and products.

Genes	S/AS	Primer Sequence (5′ to 3′)	Products (bp)
TGFβ1	S	GACCGCAACAACGCCATCT	306
	AS	GCCCTGTATTCCGTCTCCTT	
CTGF	S	CTTCTGCGATTTCGGCTCC	352
	AS	GGCTCGCATCATAGTTGGGT	
α-SMA	S	CTGCCGAGCGTGAGATTGT	485
	AS	CTTCGTCGTATTCCTGTTTGCT	
PAI-1	S	CAGATGTCTTCAGCCCTTGC	444
	AS	GAAGTCCACCTGTTTCACCAT	
18s	S	AGCAGGACTGGGAGACTACG	223
	AS	AGCAGGCTCTGGTGGGTGAT	

S: sense; AS: antisense.
